# Supplementary material for: Toll-like Receptor 2 Mediated Immune Regulation in Simian Immunodeficiency Virus-Infected Rhesus Macaques
Source: Vaccines (Basel). 2023 Dec 17;11(12):1861. doi: 10.3390/vaccines11121861 (PMC10747659; doi:10.3390/vaccines11121861)
Supplement: Supplementary file 1 [file vaccines-11-01861-s001.zip › Table S1.pdf]

Table S1. List of monoclonal antibodies used for flow cytometry assay

Antibody	Clone	Amount used (µL)	Source
Anti-human CD3	SP34-2	5	BD Biosciences, CA
Anti-human CD4	L200	3	BD Biosciences, CA
Anti-human CD8	SK1	3	BD Biosciences, CA
Anti-human CD14	M5E2	5	Biolegend, CA
Anti-human CD20	2H7	2	Biolegend, CA
Anti-NHP CD45	D058-1283	5	BD Biosciences, CA
Anti-human TLR2	TL2.1	5	Biolegend, CA
Live/dead fixable aqua stain	-	25; 1:100 dilution	Life Technologies, CA
