# Supplementary figures and images for: Toll-like Receptor 2 Mediated Immune Regulation in Simian Immunodeficiency Virus-Infected Rhesus Macaques
Source: Vaccines (Basel). 2023 Dec 17;11(12):1861. doi: 10.3390/vaccines11121861 (PMC10747659; doi:10.3390/vaccines11121861)

## Monocytes

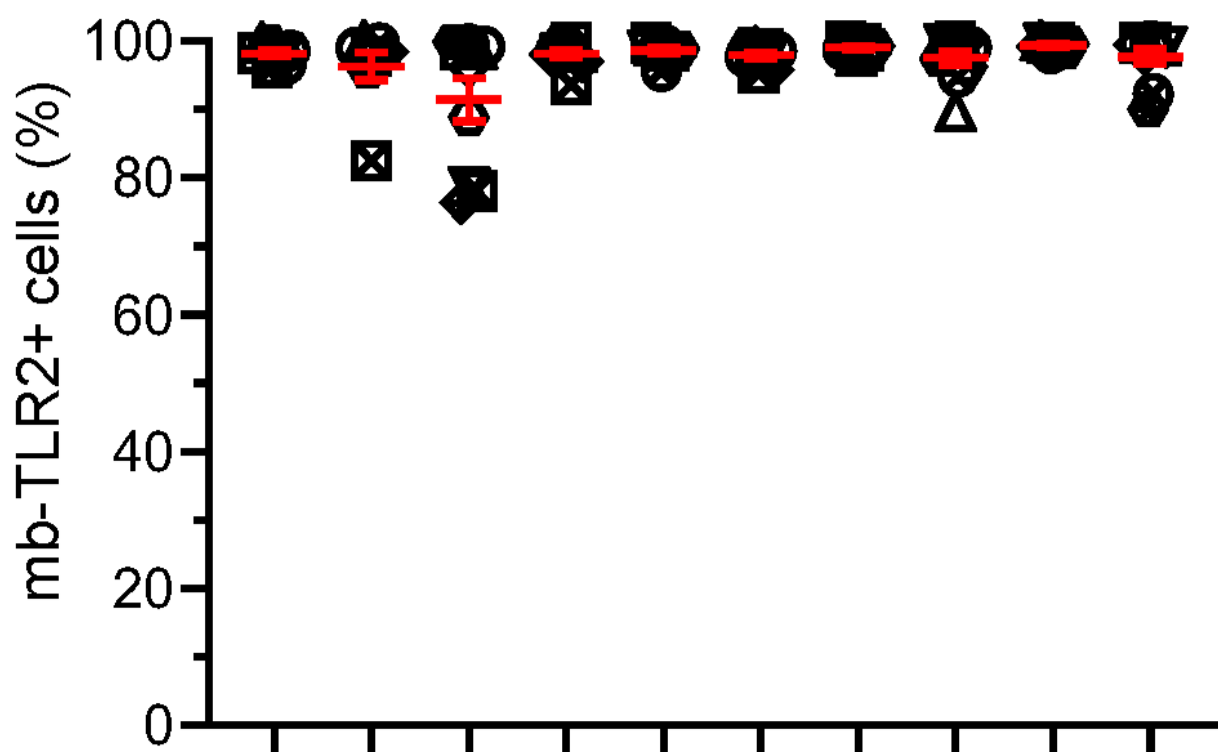

B

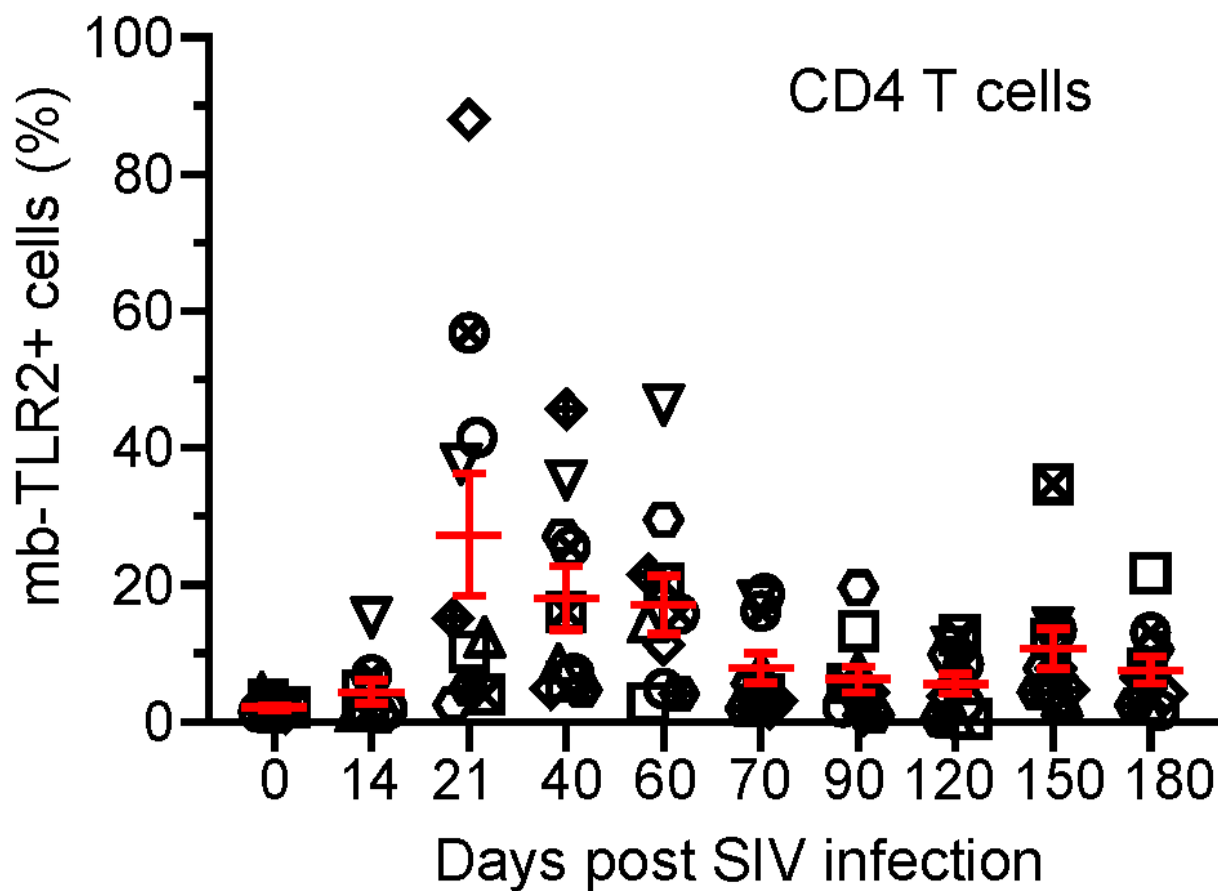

Supplement: Supplementary file 1 [file vaccines-11-01861-s001.zip › Figure S1.pdf]

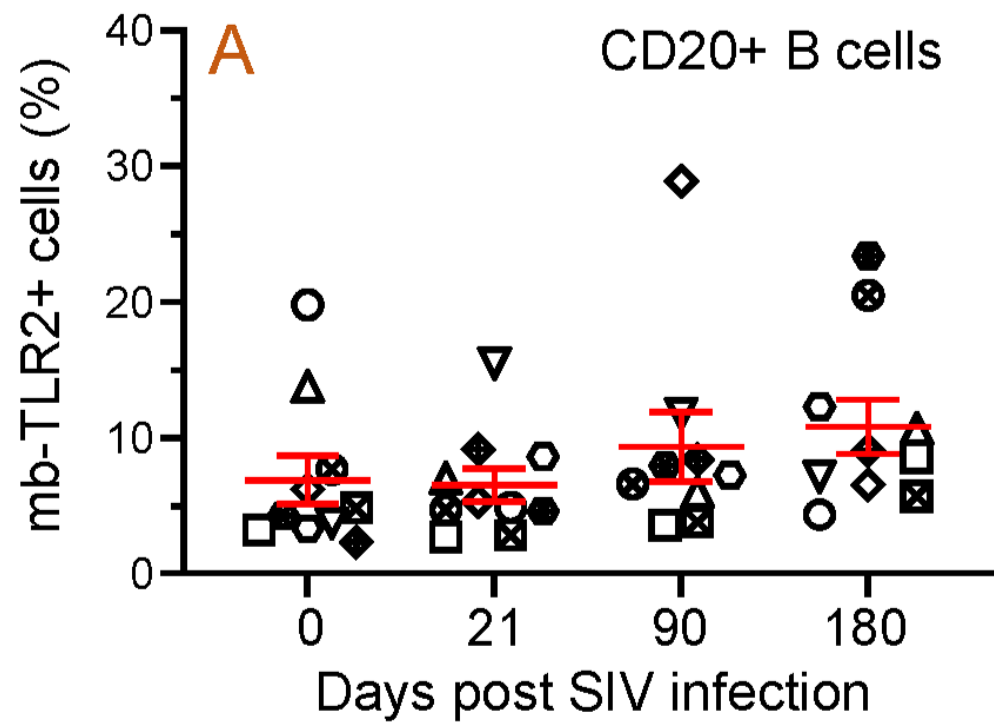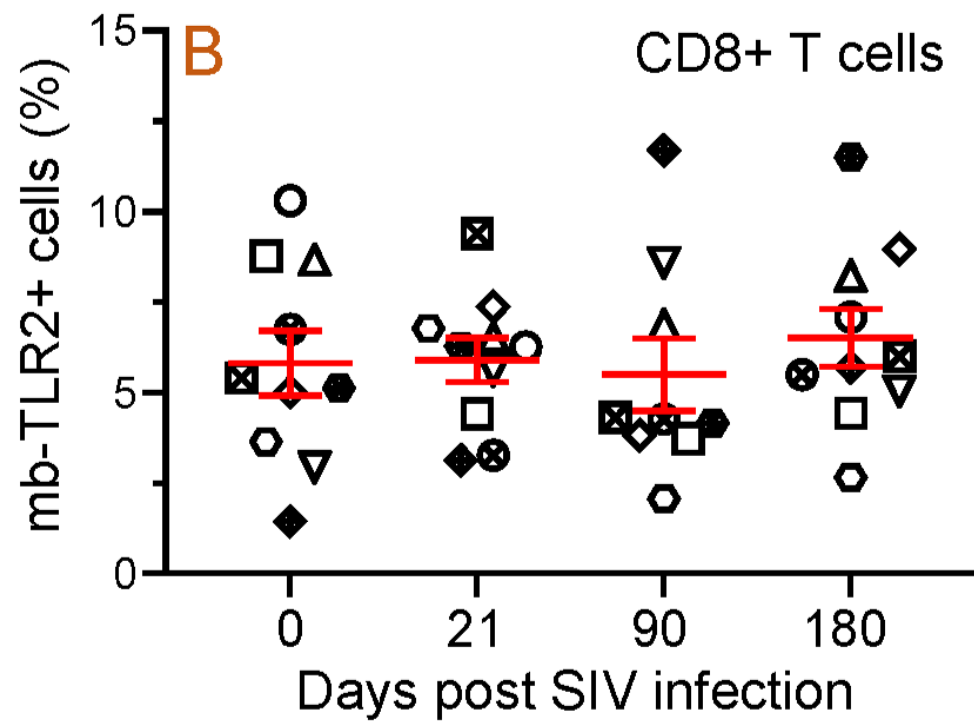

Supplement: Supplementary file 1 [file vaccines-11-01861-s001.zip › Figure S2.pdf]

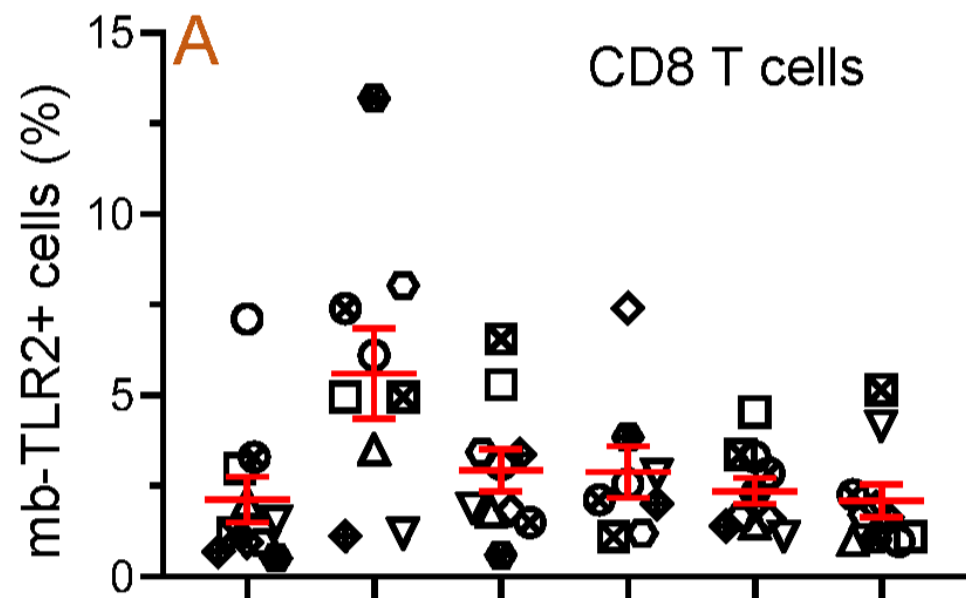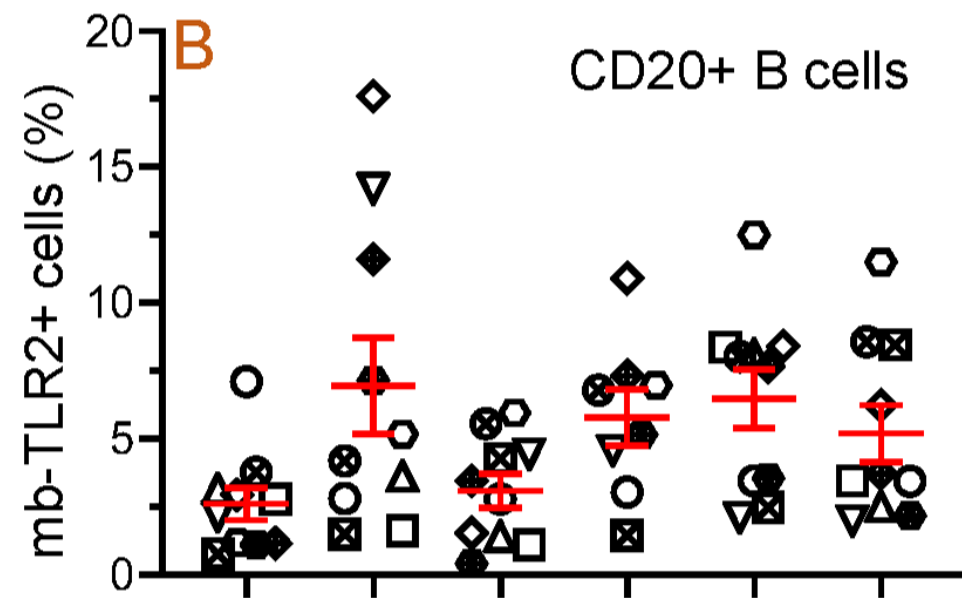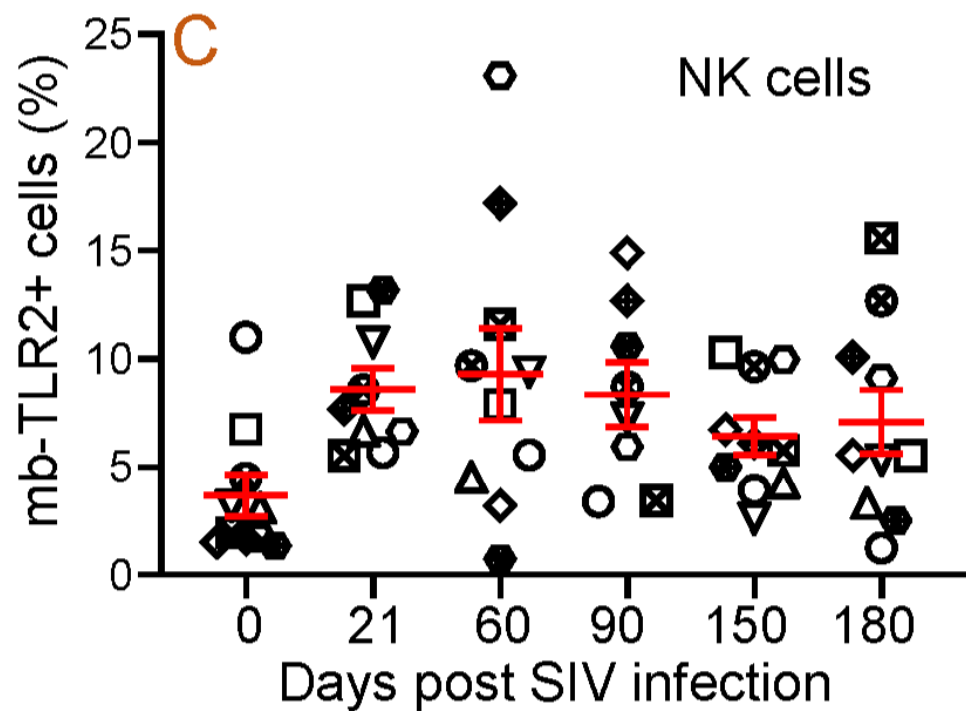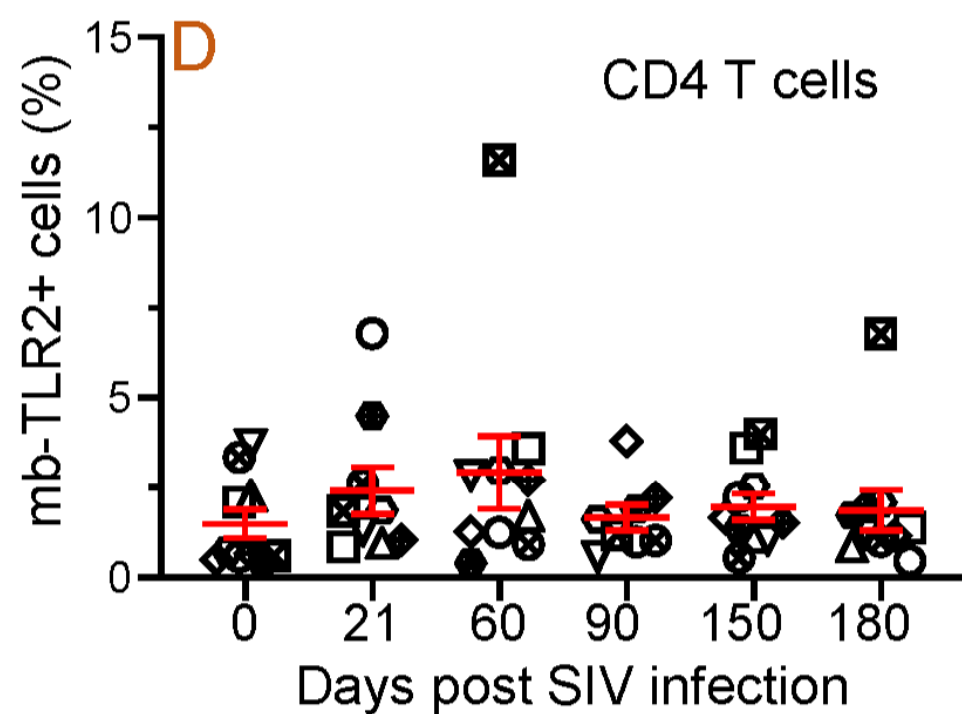

Supplement: Supplementary file 1 [file vaccines-11-01861-s001.zip › Figure S3.pdf]
